# Prisoners and cigarettes or ‘imprisoned in cigarettes’? What helps prisoners quit smoking?

**DOI:** 10.1186/1471-2458-12-508

**Published:** 2012-07-07

**Authors:** Elias Makris, Konstantinos I Gourgoulianis, Chrysi Hatzoglou

**Affiliations:** 1 Respiratory Department, University of Thessaly, Larissa 41110, Greece; 2 Physiology Department, University of Thessaly School of Medicine, Larissa 41110, Greece

**Keywords:** Quit smoking intervention, Prison, Smoking cessation centre, Imprisonment status, Smoking cellmates, Attempts to quit smoking

## Abstract

**Abstract:**

**Background:**

The aim of the study was, despite the special characteristics of prisons, to identify the features which led prisoners who attended the Smoking Cessation Centre at the Kassavetia Detention Centre in Volos (region of Thessaly, in the central part of mainland Greece) to quit smoking.

**Methods:**

Personal interviews with 204 male prisoners irrespective of smoking habitus over the period June 2008 to December 2010 were obtained. Information about medical history, history of tobacco use and addiction to narcotic use was obtained and imprisonment status was recorded. Pharmaceutical treatment (Varenicline) and counselling or only counselling were suggested as alternative strategies to them in order to help quit smoking. SPSS v15.0 software was employed, descriptive statistics were used, and a X^2^ independence test and Student’s t-test were performed.

**Results:**

Of the sample examined, 75.5% (154) were smokers. They were mainly Greeks (51.5%), single (53.4%) and had not gratuated from a high school (secondary education level) (70.6%). 59.75% begun smoking early ( ≤14 years of age ) and 64.9% were highly addicted according to Fagerstrom Tolerance Questionnaire. 74% (114) of all smokers at the prison attended the Smoking Cessation Centre. Of them, 30.7% were able to quit smoking at 3 months but 1 year later there were 20.2% ex-smokers. The key characteristics of those who were able to be ex-smokers were a change in smoking habits (decreased) compared to when free (p = .001), previous attempts to quit (while incarcerated and in general) (p = .001), average dependence levels (p < .001), started smoking after 21 years of age (p = .032), no history of addictive substance use (p = .029), being already prisoners for a longer period of time (p = .019), a limited number (3.9 ± 3.4) of prisoners per cell (p < .001) and in particular a limited number (2.8 ± 3.2) of smokers in the cell (p < .001).

**Conclusions:**

Average dependence, a past free of addictive substance abuse and a better environment of daily living for certain prisoners (as far as the number of cellmates was concerned) had a catalytic impact on prisoners finally managed to quit smoking.

## Background

The percentage of smokers internationally among special population groups such as prisoners is still high, at around 81% [1]. Smoking is one factor which causes diseases or can worsen the existing condition of health of prisoners, and with the exception of a few countries (USA, Australia), has not been adequately taken into account in relation to taking the decision to quit smoking [2,3]. Compared to the general population, the population of prisoners is disproportionably affected by health problems, including the use of addictive substances [4,5]. Detention creates obstacles in the provision of medical and hospital care in Greece either because of lack of easy access at all healthcare levels or due to staff shortages [6].

For the last 10 years, the Respiratory Department of the University of Thessaly has been testing the general population in 4 prefectures in central Greece by performing spirometry tests to diagnose chronic obstructive pulmonary disease early, and attended the Kassavetia Detention Centre in Volos with a population of 220 male prisoners for that purpose (no regulation regarding smoking). This is a low security rural prison in the prefecture of Magnesia. Quite a few of the prisoners (≈ 50) tested, stated that they wished to quit smoking permanently with medical assistance.

The high percentage of smokers in prisons and the apparently increasing number of prisoners who intended to quit smoking (expressed their wish to stop smoking on a daily basis) led to a well-planned series of measures being taken to assist this vulnerable, sensitive social group by setting up a permanent Smoking Cessation Centre at the prison.

To run a Smoking Cessation Centre for prisoners needs to consider specific aspects of closed settings: the stress [7] over lack of liberty and absence of family, the prison overcrowding [8] with smokers and non-smokers being engaged in the same cell, transfers to other prisons or court appearances [9], high degree of nicotine dependence [10] and prison leave granted in accordance with law [11].

## Methods

### Participants

The Hellenic Ministry of Justice, Department of Correctional Policy, granted ethical approval to run the Smoking Cessation Centre at the Kassavetia Detention Centre in Volos. A personal meeting with prisoners, without a guard being present, was chosen in order to protect medical confidentiality and to develop a trust-based relationship with the 204 male prisoners (smokers, ex-smokers and non-smokers) who voluntarily [12] attended the Centre over the period June 2008 to December 2010. The data was collected by research fellows and regular medical staff.

### Materials and methods

During the interview, all prisoners were informed about the harmful effects of smoking, and information was collected using questionnaires which were filled out / answered by the inmates or where it was needed by the medical staff. The methodology employed was as follows:

· A medical history was taken (age, marital status, education level, body mass index, personal medical history and number of diseases, family medical history and number of diseases, respiratory symptoms – features and duration: cough, sputum, wheezing, dyspnoea, number of respiratory symptoms, blood pressure, pulse, pharmaceutical treatment for existing diseases, visits to doctors and diagnoses based on individual health booklet).

· Data was collected regarding the use of addictive substances or narcotics (age at the onset of use, pharmacological category of substance, number of substances, attempts at quitting, duration of substance-free period).

· Imprisonment status was also recorded (reason for imprisonment, length of sentence, remaining sentence to be served, first time imprisonment or repeated offender, convicts, prisoners on remand or awaiting deportation, number of cellmates, number of cellmates who smoked, prison work, type of work and hours spent at work, whether exercise is taken during detention, hobbies in free time).

In addition, the following information was collected for smokers:

· A smoking history [age at initiation of smoking, reason for starting, pack/years, cigarettes per day over the last year, whether they combine cigarettes with something else (for example: coffee, food), use of other tobacco products, monthly expenditure on smoking, who covers the cost, whether smoking habits have changed in relation to life outside of prison, whether they want to quit smoking or not, reason for their wish to quit or not, previous attempts to quit (inside or outside prison), method / number of attempts, smoking-free period, rating on the Fagerstrom nicotine dependence scale [13].

The medicinal product used was Varenicline [14] at the recommended dose for a period of 3 months, or 4 months if required. Recommended dose in Greece:

Day 1–3: 0.5 mg once per day

Days 4–7: 0.5 mg twice per day

Days 8-completion of treatment: 1 mg twice per day

Pharmaceutical treatment using varenicline (free of charge) and counselling or counselling alone were offered to prisoners. Counselling was provided by specially trained medical staff. A 45–60 minute follow-up was carried out every week on patients during the first month of treatment and every second week in the following months to check their progress in quitting smoking. This was measured via self-reports and via CO levels measurements on every visit. During these follow-up, treatment was discussed in relation to any side-effects, how withdrawal symptoms were being dealt with, how to prevent relapse, and how to boost patient morale by rewarding their endeavours. There was no comparison of cessation rates for both treatments due to the small number of participants who preferred counseling sessions. The Smoking Cessation Centre in prison runs permanently and after the study period offers to newcoming prisoners the same choice of treatment.

### Data analysis

SPSS v15.0 software was employed, descriptive statistics were used, and a X^2^ independence test and Student’s t-test were performed. In this paper the variables are presented as the mean ± the standard deviation (SD). Results with a p <0.05 were deemed to be statistically significant.

## Results

### Sample

Of the 204 male prisoners studied 154 (75.5%) were smokers, 15 (7.35%) ex-smokers and 35 (17.15%) non-smokers. Their mean age was 33.6 ± 12.5 years.

### Demographic information

Out the 204 prisoners who attended the Centre the majority was primarily Greek (51.5%), single (53.4%) and had not finished secondary education (70.6%). The results regard the whole sample: smokers, former smokers and non-smokers. A significant percentage of smokers (22.1%) were illiterate (who do not read/write even if they attended school). Demographic data of the study in detail for every category of participants are presented in Table 1.

**Table 1:** Demographic data of total number of participants in questionnaires

**Characteristics**	**Smokers**		**Ex-smokers**		**Non-smokers**		**Total**	
	**n**	**(%)**	**n**	**(%)**	**n**	**(%)**	**n**	**(%)**
Marital status								
single	83	(53.9)	6	(40)	20	(57.15)	109	(53.5)
married	40	(26)	5	(33.3)	9	(25.7)	54	(26.45)
divorced	23	(14.9)	4	(26.7)	6	(17.15)	33	(16.15)
widowed	8	(5.2)	0	(0)	0	(0)	8	(3.9)
Education								
illiterate	34	(22.1)	1	(6.7)	2	(5.7)	37	(18.15)
primary school	42	(27.3)	3	(20)	9	(25.7)	54	(26.5)
junior high school	37	(24)	5	(33.3)	11	(31.4)	53	(25.95)
senior high school	32	(20.8)	5	(33.3)	8	(22.9)	45	(22.05)
technological college	0	(0)	0	(0)	1	(2.9)	1	(0.5)
university	9	(5.8)	1	(6.7)	4	(11.4)	14	(6.85)
Nationality								
Greek	83	(53.9)	8	(53.3)	14	(40)	105	(51.5)
Albanian	30	(19.5)	4	(26.7)	10	(28.6)	44	(21.5)
Bulgarian	12	(7.8)	0	(0)	5	(14.3)	17	(8.4)
Other nationality	29	(18.8)	3	(20)	6	(17.1)	38	(18.6)
								
	**Mean**	**S.D.**	**Mean**	**S.D.**	**Mean**	**S.D.**	**Mean**	**S.D.**
Age	33.9	13	37.5	12.2	30.9	9.7	33.6	12.5

### Imprisonment status

The mean sentence being served by prisoners who attended the Centre was 27.4 ± 36.7 months, and 77.9% had been convicted and 75% imprisoned for the first time. The mean number of cellmates who smoked was 5.8 ± 3.6 (in each cell live 6 to 12 inmates). Non-smokers shared their cell more frequently with smokers than smokers (p = <.001). Table 2 displays these results.

**Table 2:** Imprisonment status

**Characteristics**	**Smokers**		**Ex-smokers**		**Non-smokers**		**Total**	
	**n**	**(%)**	**n**	**(%)**	**n**	**(%)**	**n**	**(%)**
Detention (1)								
first-time offender	108	(70.1)	11	(73.3)	34	(97.1)	153	(75)
repeat offender	46	(29.9)	4	(26.7)	1	(2.9)	51	(25)
Detention (2)								
convicted prisoner	116	(75.3)	15	(100)	28	(80)	159	(77.95)
prisoner on remand	38	(24.7)	0	(0)	7	(20)	45	(22.05)
	**Mean**	**S.D.**	**Mean**	**S.D.**	**Mean**	**S.D.**	**Mean**	**S.D.**
length of sentence already served (months)	28.2	38.7	16.6	14.7	28.7	34.4	27.4	36.7
cellmates	6.7	3.8	5.7	4.1	7.7	3.7	6.8	3.8
smoking cellmates	5.7	3.6	4.7	3.8	6.5	3.3	5.8	3.6

### Smoking status

The smoking history of smokers attending the Centre showed that they smoked 27.8 ± 13.2 cigarettes per day. 49.4% smoked 20 or fewer cigarettes per day while 50.6% smoked over 20 (smoking habits in prison). The number of cigarettes smoked increased by 37.65% over the length of time they were in prison (prisoners who intended to quit smoking: 34.2% and prisoners who did not intend to quit:47.5%). 23.4% had made previous attempts to quit and after participating in the study that percentage augmented −74%. The vast majority (63.15%) of those who want to quit smoking had nicotine high dependence on nicotine, started smoking early in their lifes (≤14 years) and smoked less cigarettes than prisoners who did not want to quit smoking. Almost all (96.1%) smokers knew that smoking harmed their health. Table 3 displays these results.

**Table 3:** Smoking history of prisoners who intended/not intended to quit smoking

**Characteristics**	**Intention to quit smoking**		**No intention to quit smoking**		**Total**	
	**Mean**	**S.D.**	**Mean**	**S.D.**	**Mean**	**S.D.**
Cigarettes/day	26.8	13.1	30.5	13.4	27.8	13.2
Years of smoking	18.7	12.8	19.1	12.3	18.8	12.6
Pack/Years (PYS)	23.2	20	26.8	19.4	24.1	19.8
	**n**	**(%)**	**n**	**(%)**	**n**	**(%)**
Smoking initiation at						
≤14	61	(53.5)	34	(85)	95	(61.7)
15-20	40	(35.1)	2	(5)	42	(27.3)
≥21	13	(11.4)	4	(10)	17	(11)
Fagerstrom Tolerance Questionnaire						
high dependence	72	(63.15)	28	(70)	100	(64.9)
average dependence	27	(23.7)	9	(22.5)	36	(23.4)
low dependence	15	(13.15)	3	(7.5)	18	(11.7)
Smoking habits in prison						
increased	39	(34.2)	19	(47.5)	58	(37.65)
decreased	36	(31.6)	10	(25)	46	(29.9)
remained the same	39	(34.2)	11	(27.5)	50	(32.45)
Previous attempts to quit smoking	30	(26.3)	6	(15)	36	(23.4)
Current attempts to quit smoking	114	(100)	0	(0)	114	(74)
Yes, smoking harms health	112	(98.2)	36	(90)	148	(96.1)

### Factors influencing cigarette smoking and smoking cessation

The main reason reported by participants for wanting to quit smoking (a single answer was requested) was respiratory symptoms (31.6%), and for denying to quit (one or more answers) was the lack of liberty and the absence of family (100%). Lack of liberty and the absence of family (49.4%) and enforced cohabitation in the same cell with other smokers (26.5%) were the main reasons why those who attempted to quit failed to actually quit smoking. Table 4 displays these results.

**Table 4:** Reasons for intention/no intention to quit and failed attempt to quit smoking

	*Smokers	
	**n**	**(%)**
Reasons for intention to quit smoking		
respiratory symptoms	36	(31.6)
health problems	24	(21.1)
Improved physical condition	13	(11.4)
smoking dependence	11	(9.6)
other reasons	30	(26.3)
Reasons for no intention to quit smoking		
lack of freedom and the absence of family	36	(90)
use of nicotine to reduse stress	22	(55)
smoking dependence	14	(35)
Reasons for failing to quit smoking		
lack of freedom and the absence of family	41	(49.4)
enforced cohabitation in the same cell with other smokers	22	(26.5)
smoking depedence	6	(7.2)
use of nicotine to reduse stress (as reported by prison inmates)	14	(16.9)

* total number of participants in the cessation program.

### Treatment interventions

Of the 114 smokers who intended to quit smoking (74%), majority choose the combined treatment. More specifically 98 (86%) received Varenicline treatments and counselling, 29 (29.6%) completed the treatment and another 16 (14%) only received counselling as was their wish. 35 (30.7%) managed to quit smoking at the end of 3 months of treatment (varenicline and counselling:27, counselling:8) and 1 year later there were 23 ex-smokers (20.2%) (varenicline and counselling:19, counselling:4). Table 5 displays these results.

**Table 5:** Quit smoking intervention

**Treatment**	**Intention to quit smoking**		**Initial cessation**		**Ex-smokers**	
	**n**	**(%)**	**n**	**(%)**	**n**	**(%)**
Varenicline and counselling	98	(86)	27	(27.55)	19	(19.4)
counselling	16	(14)	8	(50)	4	(25)
TOTAL	114	(100)	35	(30.7)	23	(20.2)
Integrated treatment	29	(29.6)	19	(70.4)	17	(62.3)

### Quit smoking characteristics

Statistical analysis correlated characteristics which encouraged those prisoners (n = 23) who were able to quit smoking (ex-smokers: did not smoke for at least a year) achieve their goal and set them apart from those who did not achieve to quit smoking. These sets of characteristics related to (a) smoking history, (b) previous use of addictive substances and (c) the special features of their life in prison. The first set indicates that ex-smokers started smoking after 21 years of age (p = .032), had average levels of dependence (p < .001), reduced levels of smoking while in prison (p = .001), smoked 20.2 ± 8.5 cigarettes smoked per day (p = .006) and previous attempts to quit (p = .001). Table 6 displays these results. The second set shows that ex-smokers showed less substance abuse in the past) (p = .029). Table 7 displays these results. The third set included the small number of cellmates (p < .001), the small number of smokers in the cell (p < .001) and the greater length of the sentence (p = .019), all characteristics which differed for those prisoners who did not manage to quit smoking. Table 8 displays these results.

**Table 6:** Characteristics of smoking history that helped participants quit smoking

**Characteristics**	**p-value**	**Ex-smokers**		**Smokers**		**Total**	
		**n**	**(%)**	**n**	**(%)**	**n**	**(%)**
Age when they started smoking							
≤14	ns	7	(30.45)	54	(59.3)	61	(53.5)
15-20	ns	11	(47.8)	29	(31.9)	40	(35.1)
≥21	.032	5	(21.75)	8	(8.8)	13	(11.4)
Fagerstrom Tolerance Questionnaire							
high dependence (7–10)	ns	6	(26.1)	66	(72.5)	72	(63.15)
average dependence (4–6)	<.001	13	(56.5)	14	(15.4)	27	(23.7)
low dependence (0–3)	ns	4	(17.4)	11	(12.1)	15	(13.15)
Smoking habits in prison							
increased	ns	7	(30.4)	32	(35.15)	39	(34.2)
decreased	.001	14	(60.9)	22	(24.15)	36	(31.6)
the same	ns	2	(8.7)	37	(40.7)	39	(34.2)
Previous attempts to quit smoking							
no	ns	10	(43.5)	74	(81.3)	84	(73.7)
yes	.001	13	(56.5)	17	(18.7)	27	(26.3)
							
		**Mean**	**S.D.**	**Mean**	**S.D.**	**Mean**	**S.D.**
Cigarettes/day	.006	20.2	8.5	28.5	13.6	26.8	13.1

**Table 7:** Characteristics of past use of addictive substances that helped participants quit smoking

**Characteristics**	**p-value**	**Ex-smokers**		**Smokers**		**Total**	
		**n**	**(%)**	**n**	**(%)**	**n**	**(%)**
Use of addictive substances							
no	.029	19	(82.6)	51	(56)	70	(61.4)
yes	ns	4	(17.4)	40	(44)	44	(38.6)

**Table 8:** Characteristics during life in prison help participants quit smoking

**Characteristics**	**p-value**	**Ex-smokers**		**Smokers**	
		**Mean**	**S.D.**	**Mean**	**S.D.**
Number of cellmates	<.001	3.9	3.4	7.3	3.5
Number of smokers in cell	<.001	2.8	3.2	6.3	3.2
Length of sentence (months)	.019	49.7	46.5	26.6	40.2

## Discussion

### Smoking cessation interventions in correctional systems

This is the first time a permanent prison Smoking Cessation Centre has been set up and run in Greece and one of the few worldwide. Studies conducted abroad have primarily focused on smoking habits among prisoners and the factors affecting them, their desire to quit smoking and have only outlined recommendations about how to intervene with this special population group so as to help them quit smoking. There have been few interventions in prisons (mainly women’s prisons) involving psychotherapy and nicotine substitutes. Moreover, international studies [10,15] have also been carried out at prisons where total or partial smoking bans have been in place, and have dealt with prisoner (and employee) compliance with the ban, and nicotine withdrawal symptoms No such study has been carried out in Greece.

This well-planned intervention from the Respiratory Department of the University of Thessaly, involving the establishment of a Smoking Cessation Centre at the prison, was the stimulus and incentive that led both active and passive smokers to visit the Centre. Everyone was informed about the effects of smoking, about the benefits of quitting smoking, and a medical history was undertaken. Furthermore, medical consultation was provided to anyone who asked for it.

### Correctional populations and differences compared to the general population

This is the first study in Greece on the smoking habits of the prison population and it showed several differences compared to the general population. It has been reported that Greeks (especially Greek men) are in the first position at European and worldwide studies in terms of the number of smokers, with the figure close to 49.9% [16]. 51.5% of the prison population studied were Greeks and we observed that the percentage of smokers is even higher (75.5%), which is in accordance with studies in the international literature on similar populations [17], with a quite low smoking index for the general population [18]. In other words, smoking habits among the prison population are independent (different) compared to that of the general population, but always high.

This finding is bolstered by the characteristics of smokers in the general population identified by a recent study which found that 42.7% have graduated a university and 32.9% started smoking between the ages of 15 and 18 [19], while 76.2% of smokers in prison had not completed education at school (secondary level) [20] and 62.5% had started smoking before the age of 14 years.

### Special characteristics of prisoners

Factors such as (a) the lack of liberty and the absence of family resulting in intense stress, (b) prison over-population resulting in smokers and non-smokers sharing the same cell, without attempts being made to segregate them, (c) transfers to other prisons (either scheduled or for security reasons) or court appearances (for hearings – appeals or to give testimony), (d) nicotine dependence (and use of nicotine to reduce stress and help prisoners front their problems as reported by prison inmates) and (e) leave that certain prisoners are entitled to by law (some are not entitled to leave), during which they are in a completely different environment from that of the prison, because they are temporarily free, are factors which cause a worsening of or return to smoking habits among prisoners who smoke, whether they are attempting to quit or not. In other words prisoners who are trying to quit are relapsing and those who do not quit increase their use. Transfers and court appearances in particular interrupt medical follow up and they may cause relapses. Replies to the relevant questions during the interview clearly showed those factors which need to be taken into account to any intervention taken or planned in relation to this special population. Ignoring the nicotine dependence factor, which also exists among the general population, the other factors are different for and representative of and characterise the prison population.

To ensure that the prison Smoking Cessation Centre operated without problems and that certain of those factors were eliminated, it was -and is- necessary for there to be frequent communication with the patients themselves and with the administration of the prison, and so far the results have been excellent. In this way transfers and leave (which are situations which change the daily habits of prisoners trying to quit smoking) are covered. Medical and counselling support continued to be provided in these cases so that there were no gaps or periods that patients were 'missing' from medical supervision. From the outset prison management was a valuable aid in running the Smoking Cessation Centre. Moreover, it is worth noting that the existence of the Centre at the prison was an incentive for both administrative staff and prison guards to quit smoking (staff and guards was not included in the program).

## Conclusions

In conclusion, the Smoking Cessation Centre at the Kassavetia Detention Centre in Volos may be an important factor in improving the quality of life of prisoners for the remainder of their sentence and when they rejoin society by acting a) in a preventative - dissuasive manner by informing smokers, ex-smokers and non-smokers about the harmful effects of active and passive smoking and about the benefits of quitting smoking and b) in a therapeutic manner by administering Varenicline to smokers (with their consent) and by providing counselling.

The smoking cessation results of those persons who attended the Smoking Cessation Centre were particularly significant both initially after the end of treatment and in terms of final smoking cessation, especially when one takes into consideration the special conditions in prisons and the fact that smoking tends to increase by 34.2% among prisoners over the course of their sentence. The fact that after the Centre opened 73.4% of prisoners attempted to quit, whereas before the Centre was established only 23.4% had attempted to quit, is also a success. Average dependence, a past free of addictive substance abuse and a better environment of daily living for certain prisoners (as far as the number of cellmates was concerned) had a catalytic impact on prisoners finally managed to quit smoking.

## Competing interests

The authors declare to have none competing interests.

## Authors’ contribution

Author EM wrote the protocol, conducted the statistical analysis and wrote the first draft of the manuscript and all authors contributed to and have approved the final manuscript. Author KIG designed the study. Author CH conducted literature searches and provided summaries of previous research studies.

## Pre-publication history

The pre-publication history for this paper can be accessed here:

http://www.biomedcentral.com/1471-2458/12/508/prepub
